# A review of substitute CT generation for MRI-only radiation therapy

**DOI:** 10.1186/s13014-016-0747-y

**Published:** 2017-01-26

**Authors:** Jens M. Edmund, Tufve Nyholm

**Affiliations:** 1 Radiotherapy Research Unit, Department of Oncology, Herlev & Gentofte Hospital, Copenhagen University, Herlev, Denmark; 2 Niels Bohr Institute, Copenhagen University, Copenhagen, Denmark; 3 Department of Radiation Sciences, Umeå University, Umeå, SE-901 87 Sweden; 4 Medical Radiation Physics, Department of Immunology, Genetics and Pathology, Uppsala University, Uppsala, Sweden

## Abstract

Radiotherapy based on magnetic resonance imaging as the sole modality (MRI-only RT) is an area of growing scientific interest due to the increasing use of MRI for both target and normal tissue delineation and the development of MR based delivery systems. One major issue in MRI-only RT is the assignment of electron densities (ED) to MRI scans for dose calculation and a similar need for attenuation correction can be found for hybrid PET/MR systems. The ED assigned MRI scan is here named a substitute CT (sCT). In this review, we report on a collection of typical performance values for a number of main approaches encountered in the literature for sCT generation as compared to CT. A literature search in the Scopus database resulted in 254 papers which were included in this investigation. A final number of 50 contributions which fulfilled all inclusion criteria were categorized according to applied method, MRI sequence/contrast involved, number of subjects included and anatomical site investigated. The latter included brain, torso, prostate and phantoms. The contributions geometric and/or dosimetric performance metrics were also noted. The majority of studies are carried out on the brain for 5–10 patients with PET/MR applications in mind using a voxel based method. T1 weighted images are most commonly applied. The overall dosimetric agreement is in the order of 0.3–2.5%. A strict gamma criterion of 1% and 1mm has a range of passing rates from 68 to 94% while less strict criteria show pass rates > 98%. The mean absolute error (MAE) is between 80 and 200 HU for the brain and around 40 HU for the prostate. The Dice score for bone is between 0.5 and 0.95. The specificity and sensitivity is reported in the upper 80s% for both quantities and correctly classified voxels average around 84%. The review shows that a variety of promising approaches exist that seem clinical acceptable even with standard clinical MRI sequences. A consistent reference frame for method benchmarking is probably necessary to move the field further towards a widespread clinical implementation.

## Introduction

**Fig. 1:**
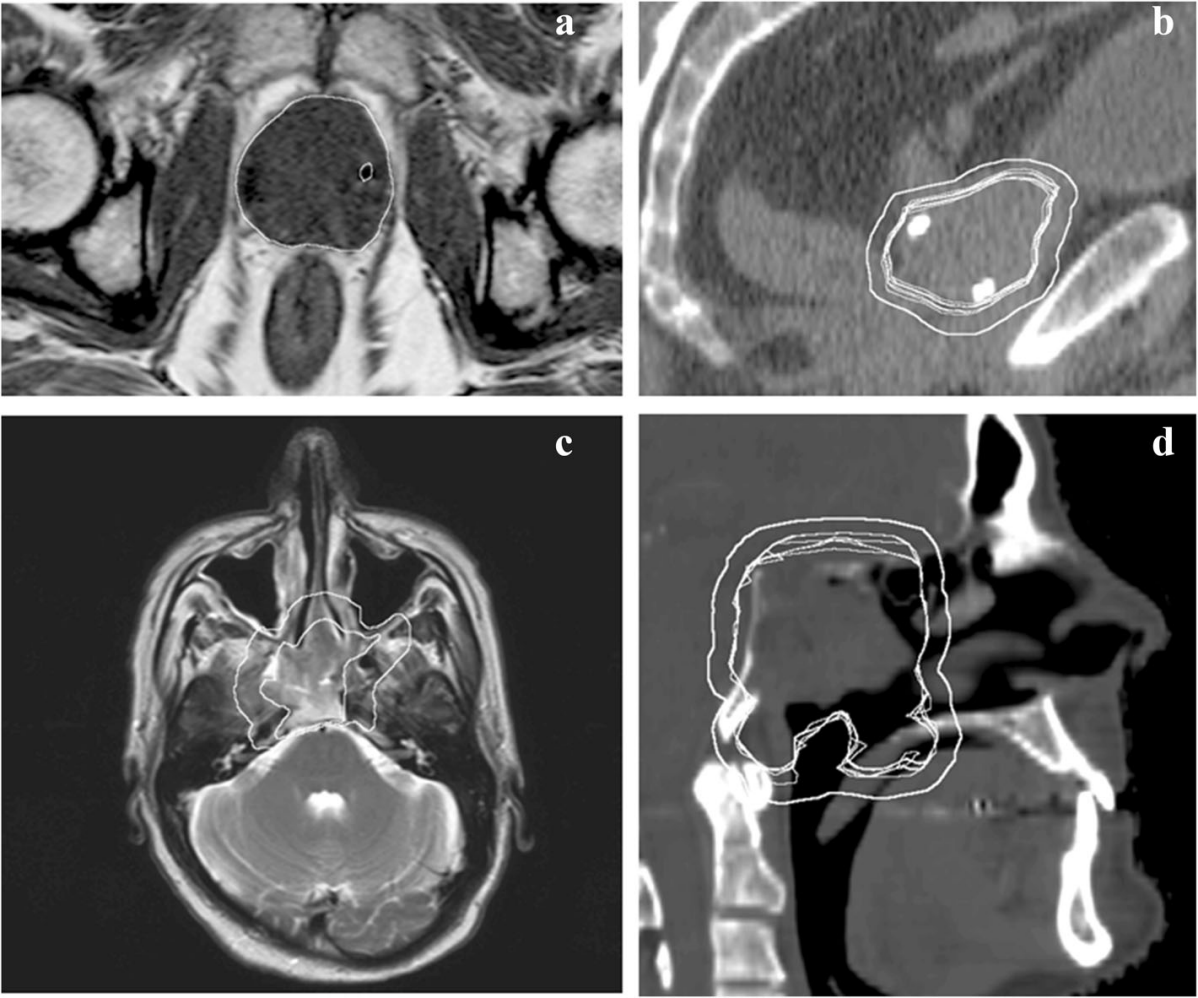
Variability of multiple registrations between MRI and the corresponding CT for prostate (*top*) and nasopharynx (bottom). **a**: One marker (of three) indicated by a white circle on the axial MRI. **b**: Two markers shown by the *white dots* on the sagittal CT. The multiple thin *white lines* are the MRI delineated clinical target volume (CTV) transferred to the CT based on the marker registration following department protocol from 7 different observers. The protocol is based on a rigid automatic (mutual information) registration for a limited FOV around the prostate followed by a manual adjustment to match the markers in the three planes. The outermost white line was the planned target volume (PTV) applied. The data are taken from reference [59]. **c**: The gross target volume (*inner*) and CTV (*outer*) as delineated on the axial MRI. **d**: The multiple thin *white lines* are the MRI based CTV transferred to the CT by 6 different observers (sagital CT slice shown). The registration is based on a rigid automatic (mutual information) registration followed by a manual fine adjustment. The outermost *white line* was again the applied PTV. The data are taken from reference [60]

Dose calculations performed on scans from magnetic resonance imaging (MRI) were first reported around the millennium when MRI emerged as a complimentary modality to computed tomography (CT) in the delineation step of the radiotherapy (RT) chain [1, 2]. As MRI provides superior soft tissue contrast and delineation precision as compared to CT [3–8], the concept of carrying out all steps of the RT chain on MRI as the sole modality, so-called MRI-only RT, could provide a favorable workflow. MRI-only RT would further remove a systematic registration error when transferring MRI delineated structures to the CT which has been reported to be in the order of 2–5 mm for various treatment sites [9–13]. As CT is used for positioning of the patient at treatment, registration errors introduce a spatial systematic uncertainty. The dosimetric impact of a systematic error will increase when the radiation is aimed at small structures or when the target is close to sensitive organs. This could be the case for small tumors or the hippocampus in the brain [14] with a structure radius in the order of a possible registration error or when a standard PTV or PRV margin has to be compromised to maintain an acceptable therapeutic ratio. An example of the MRI to CT registration variability for a prostate and nasopharynx case is illustrated in Fig. 1.

In addition, MRI-only will decrease the number of scans and associated patient discomfort, and, reduce the planning related costs [15]. The benefits of MRI-only RT would further increase in a workflow with repeated imaging, e.g. weekly scans, for response assessment and/or treatment adaptation.

A number of concerns related to MRI-only RT exist. One major challenge of performing dose calculations on MRI is the lack of correspondence between the voxel intensity and the associated attenuation property of the tissue. Unlike CT images where the voxel intensity directly reflects the radiological characteristics of the tissue, MRI intensities rather correlate with tissue proton density and the magnetic relaxation, i.e. the inertia of the dipole moment [16]. This leads to voxel ambiguity for tissues such as bone and air which both appear dark on the MRI although they have very different attenuation coefficients. The focus of this review is strategies for dealing with this ambiguity. Further challenges constitute scanner induced geometrical distortions arising from gradient non-linearity and magnet inhomogeneities and patient induced artifacts such as susceptibility and chemical shifts [13]. Specific problems for algorithms converting the MRI signal into a CT number further constitute normalization of absolute signal intensities and data correction strategies such as bias field correction. These topics are considered out of scope of this review.

**Fig. 2:**
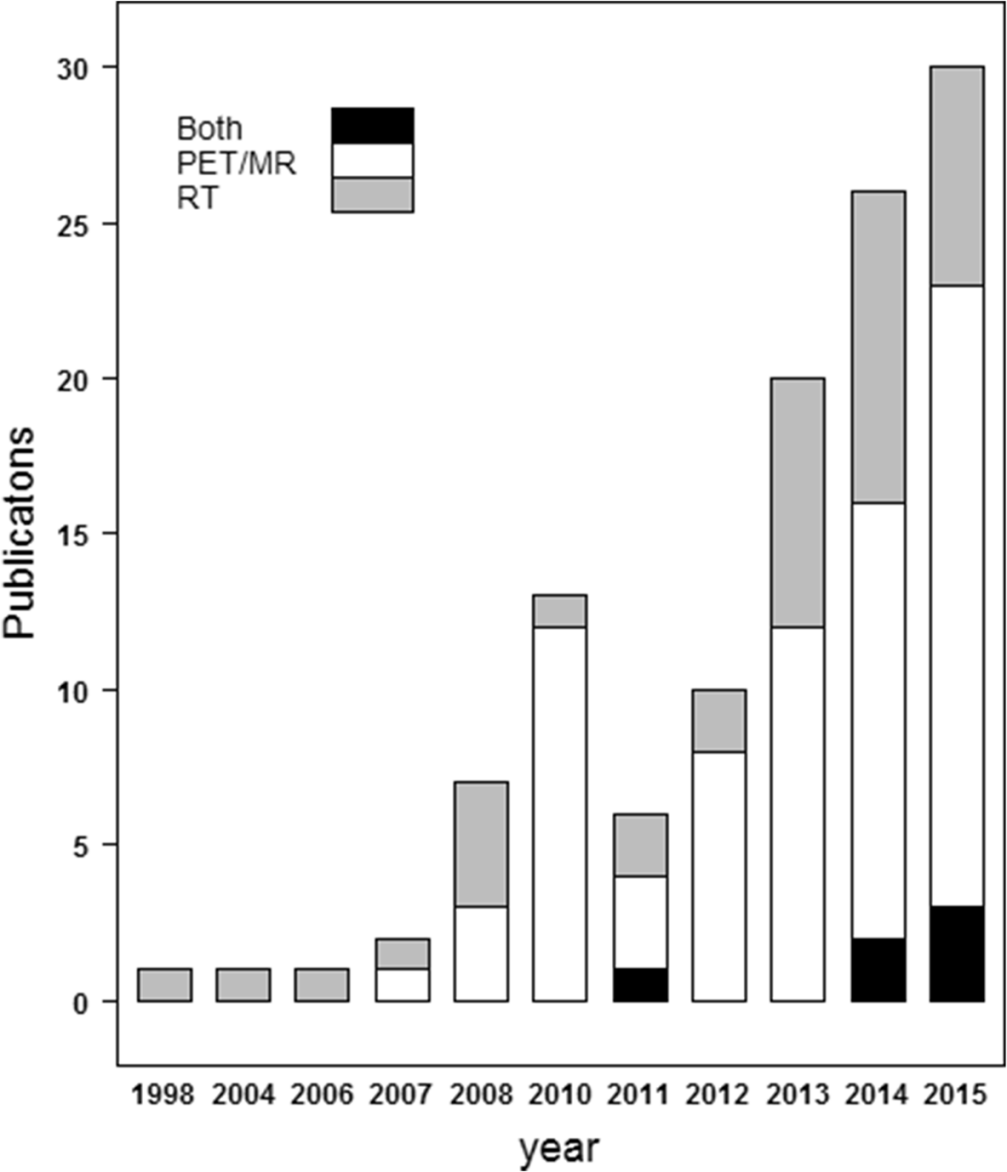
The number of articles versus time after applying the search string and exclusion criterion 1 provided in the text. The publications are grouped into proposed applications of the described method and sorted according to publication year. A boost in the amount of publications can be inspected around 2010 (PET/MRI) and 2013-14 (MRI guided EBRT)

The increased use of MRI for target and normal tissue delineation in RT in general and two device-driven events have facilitated scientific activity for assigning electron densities to MRI images^1^. The first event is the commercial availability of clinical integrated hybrid PET/MRI systems around 2010-2011 [17]. Unlike traditional PET/CT systems where the CT scan is used for attenuation correction of the PET signal needed in quantitative PET volume estimates such as the standard uptake volume (SUV), attenuation coefficients need to be assigned to the MRI scan in hybrid PET/MRI systems to make a similar attenuation correction. The second event is the commercial availability of integrated MRI guided systems in external beam RT around 2014 [18]. These systems can provide MRI scans for patient setup based on soft tissues and monitor the tumor movement during treatment delivery. The systems would be able to calculate and adapt the dose distribution at each given fraction if a dose calculation can be performed on the MRI scan which requires electron densities to be assigned. The increased focus on MRI in RT and the introduction of the imaging and treatment devices is reflected in the number of publications as illustrated in Fig. 2.

In this review, we will report on a collection of typical performance values for a number of main approaches encountered in the literature for conversion of MR data to electron density or HU maps relevant for RT. The generated map is called “synthetic CT”, “substitute CT”,”pseudo CT” or similar, i.e. no common terminology is currently established. In the following, we will use the term substitute CT (sCT) since the acronym for pseudo CT (pCT) is often used for the planning CT in adaptive RT studies [19, 20]. This field of research covers both diagnostic and therapeutic radiology as well as MRI. Further, the research field expands into automatic organ segmentation and image analysis in general. As a result, a diverse amount of approaches with different scientific traditions, terminology and endpoints in mind have been reported in the literature. Consequently, we have had to make simplifications and compromise details in order to preserve an overview and to further categorize and score the methods with the aim of providing results relevant for RT. Therefore, a direct comparison between the reported performance metrics presented here is not valid. Rather, the idea is to provide an overview of the multiple strategies investigated in the literature along with a general order of accuracy that can be reached.

We have categorized the investigated methods into three main approaches. These are termed voxel [21–23], atlas [24, 25] and hybrid [26, 27]. The latter use a combination of the voxel and atlas based approach. Similar approaches are typically categories as segmentation, sequence/image contrast, template and atlas in PET/MRI terminology [16, 28]. The voxel based approach primarily uses information about voxel intensities (contrasts) in the MR images to assign electron densities. No or limited information about the location of voxels is included in this category. The voxel based methods are dominated by the concept of machine learning in which part of the data is used to train (optimize) a model which is then applied on the remaining MRI data to predict the CT numbers. If a study contains *n* patients, this is usually done by training the model on *n-1* patients and then predict the sCT on the remaining patient in a rotating scheme known as leave-one-out cross validation. The electron density assignment (CT number) can be made on the basis of generic values (e.g. from ICRU report 46 [29]) to bulk groups of voxels. Alternatively, the assignment can happen on a continuous scale by including patient specific CT numbers in a training phase. In contrast, the atlas based approaches focus on aligning the location of a patients MRI voxels to the corresponding location of a MRI voxels in an atlas through registration. The atlas can either be a single or average (template) patient or contain a number of patients (often termed multi-atlas). The atlas contains a pre-known correlation between the MRI voxels and the value of interest, e.g. CT number or organ label. Once the alignment has taken place, the atlas CT number can be assigned to the patients’ MRI scan and hence converting it into a sCT scan.

A large number of MRI sequences/contrasts for electron density assignment have been reported in the literature. We have chosen to divide the MR input images into four main contrasts / sequences categories. The first two categories are simply termed T1 weighted (T1w) and T2 weighted (T2w). They are based on common clinical MRI sequences which rely on either the longitudinal (T1) or the transverse (T2 or T2*) tissue relaxation to produce image contrast. The T1 and T2 relaxation is determined from multiple refocusing pluses during the repetition time while T2* describes the relaxation of the free induction decay (FID) produced in the receiver antenna coil. Two main pulse sequences exist for MR image acquisition: spin echo (SE) and gradient echo (GE). The SE MR signal intensity is roughly proportional to ρ[1-exp(TR/T1)]exp(-TE/T2) where ρ (proton density), T1 and T2 are tissue properties and TR (repetition time) and TE (echo time) are sequence parameters. The equation is only valid if TR > > TE which is usually the case, and, in general T1 > T2 > T2* relaxation [30]. T1w images (short TR, short TE) are preferred for visualizing anatomy while T2w images (long TR, long TE) are usually the choice for visualizing pathology. The third category comprises the Dixon family of fat-water separating sequences and is collectively termed Dixon [31]. It is based on the chemical shift between the resonance frequencies of fat and water and can be weighted towards T1, T2 or ρ as it is (typically) a SE sequence. The fourth category of MRI sequences is based on dual ultrashort echo time (dUTE) to visualize solid structures with a very short T2 relaxation time such as the bone [32, 33]. In dUTE image acquisition, a first signal is collected right after the excitation, and a second using the GE technique at a longer nominal echo-time. The first image is ρ weighted or T1w depending on the flip-angle and the second will have a T2*w or T1w contrast depending on the echo-time and the flip-angle. T2* is only possible to realize with gradient echo sequences.

## Material and methods

### Literature search

This review reports on a collection of typical performance values for substitute CT generation rather than giving a detailed theoretical background of the methods used to predict substitute CT. To give a fair representation on the scientific activity within this field, we performed a literature search in the Scopus database November 2015 [34]. Index terms such as Medical Subject Headings (MeSH) terms were not used to define a search due to the wide diversity of sciences involved in the research field and the consequent lack of common terminology. Instead, a collection of common keywords found in a number of MRI-only RT and PET/MRI articles were organized in a logical search string defining the inclusion criteria:

- TITLE-ABS-KEY [(“PET MRI” OR “MR PET”) AND NOT (functional OR diffusion OR fdg-spect)]

OR

- TITLE-ABS-KEY [(radiotherapy OR “radiation therapy”) AND (“magnetic resonance imaging” OR “magnetic resonance” OR mri OR mr) AND NOT chemotherapy]

AND

- TITLE-ABS-KEY [“Attenuation correction” OR “computed tomography substitute” OR “substitute CT” OR “pseudo CT” OR “MRI only” OR “MRI alone”]

where TITLE-ABS-KEY indicate either the title, abstract or keywords of the paper. This resulted in 254 papers and we further added 7 papers/abstracts that for various reasons were not found in the structured literature search (e.g. strange keywords, conference abstracts etc.). Three exclusion steps were then introduced. Exclusion 1 was defined as papers having TITLE-ABS-KEY on the following:

- Diagnostics and delineations based on MRI only.
- Brachytherapy
- CT to MRI registration error/IGRT studies
- Subjects specific for PET/MRI: field-of-view (FOV) truncation, effects of headphone and coils, etc.
- PET/MRI specific corrections: time-of-flight, line source, maximum likelihood for attenuation and activity (MLAA).

The application of exclusion 1 reduced the number of papers to 117. These included relevant investigations for assigning electron densities to MRI scans for the purpose of PET attenuation correction, MRI-only RT or both (see Fig. 2). Exclusion 2 intended to only include studies which presented novel methods for electron density assignment. Abstracts and manuscripts including the following were excluded:

- Review articles
- Book series or only insufficient abstracts available.
- RT feasibility/comparative studies: Dose calculations incl. and excl. CT transferred structures such as bone and air cavities to the MRI. CT bulky assigned density vs. normal CT etc.
- PET/MRI feasibility/comparative studies: Difference in SUV or similar by applying CT based vs. MRI corrected attenuation maps incl. or excl. CT transferred structures.
- Focus on MRI artifacts and distortion quantifications and corrections.
- MRI-only based workflow descriptions.

**Fig. 3:**
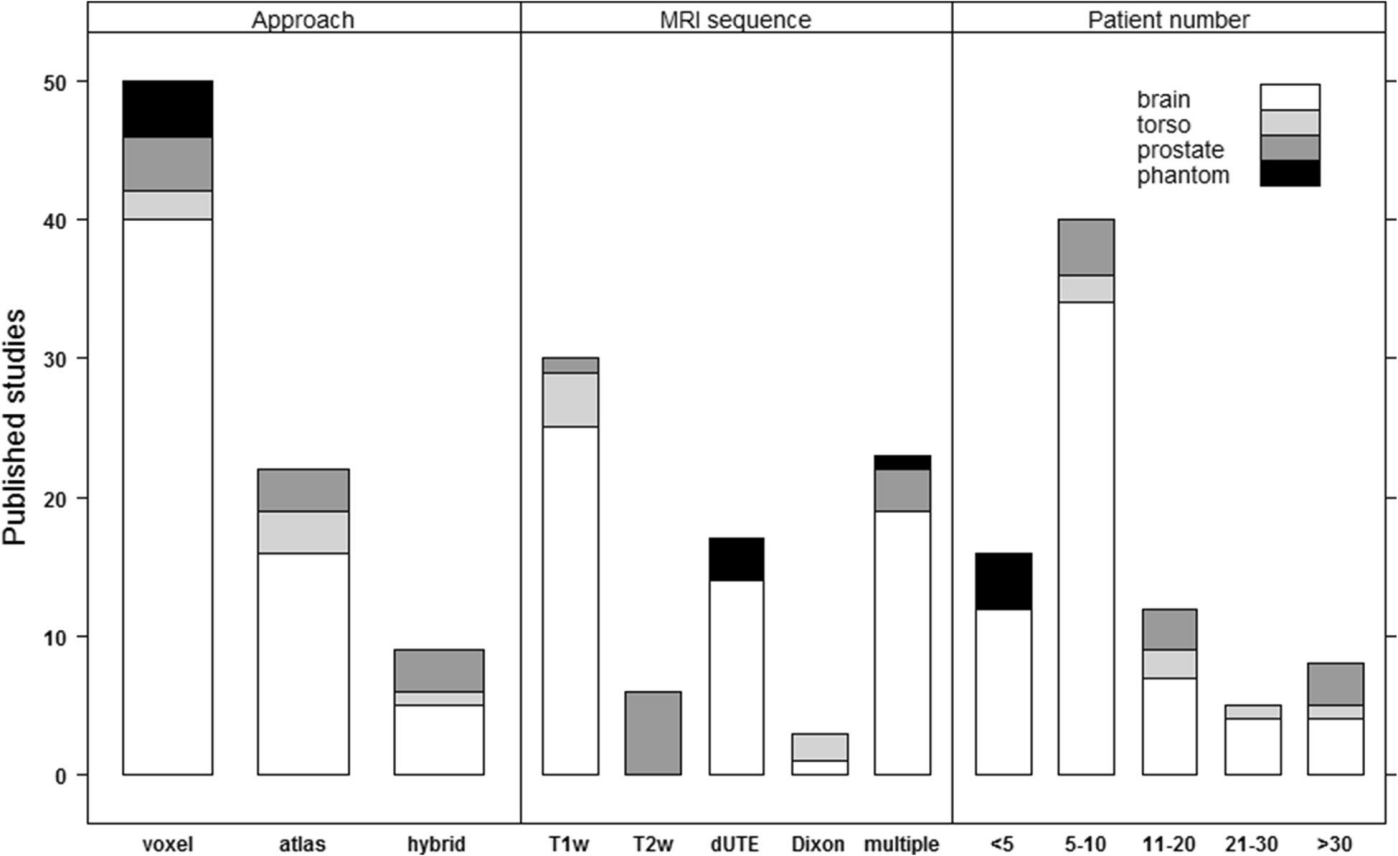
Categorization of contributions after applying exclusion criterion 2. The papers were sorted according to their main method (*left*), used MRI sequences/contrasts (*middle*) and number of subjects, i.e. phantom, patients or volunteers (*right*). In the above categories, the following simplifications were made: head = brain, whole body = torso, cervix = prostate (only 1 study), UTE = dUTE, and water/fat separating MRI sequences = Dixon. Volunteers and phantoms were categorized as patients. Some papers included description of multiple methods which were included in the histograms as separate studies, hence the term “published studies” for the ordinate

After exclusion 2, 73 papers presenting novel correction methods remained (see Fig. 3). Whenever multiple method approaches and /or MRI sequences were used these were collectively categorized as “hybrid” and “multiple”, respectively.

Exclusion 3 intended to only consider studies which included a quantitative performance metric of the resulting sCT scan which would be relevant or applicable for RT purposes. The following papers were excluded:

- No reported quantitative performance metric.
- Reported performance metric not relevant to MRI-only RT, e.g. differences in SUV, linear correlation coefficients of activity estimates, etc.

**Fig. 4:**
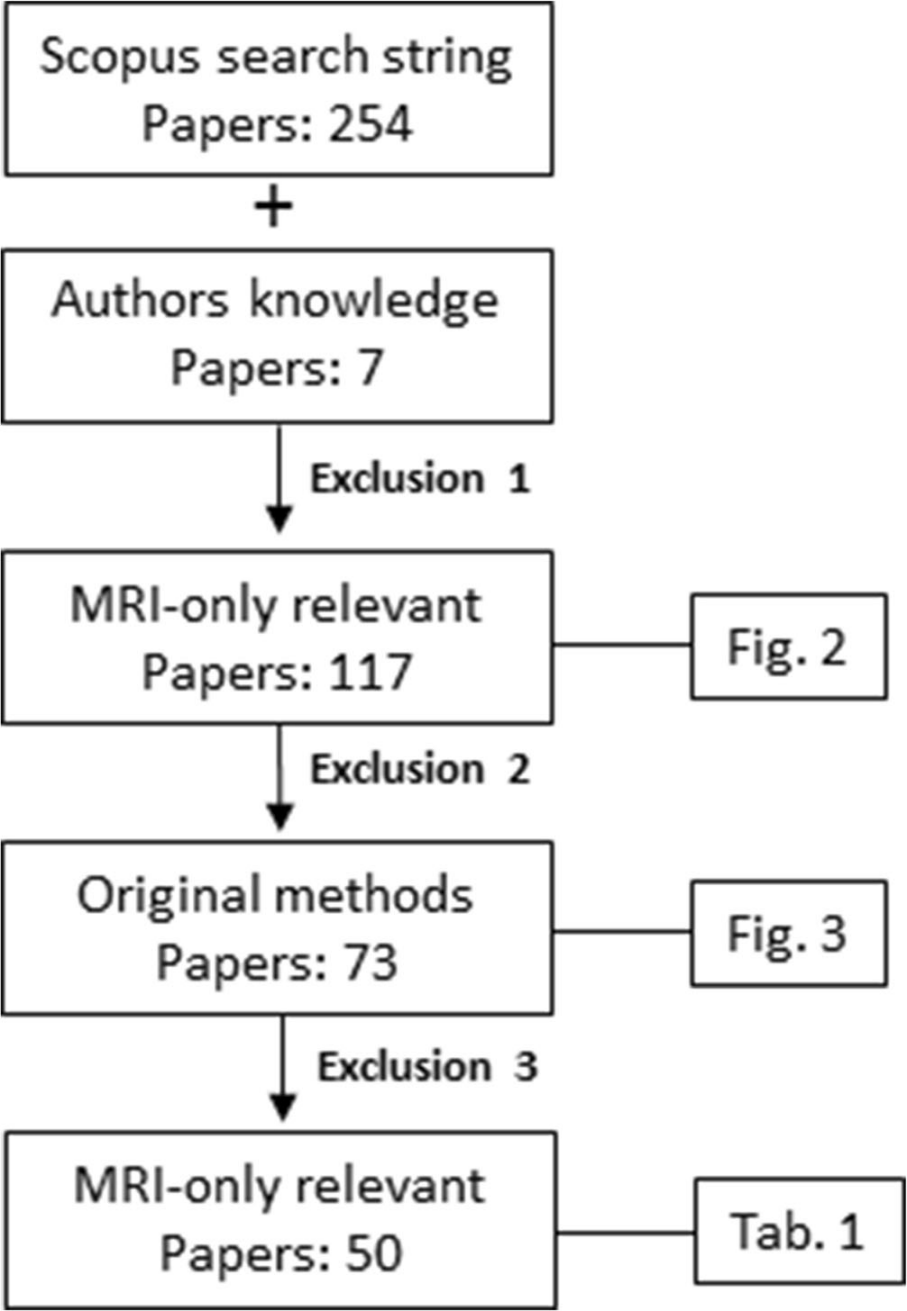
The adopted strategy for inclusion of papers with reported metrics in this review

The final 50 papers are shown in Table 1 and the selection process is summarized in Fig. 4. The final papers were arranged in the main categories as described in the introduction and further subcategorized within each main approach and applied MRI sequences when possible. The latter was limited to the four overall sequence categories.

**Table 1:** Table summarizing performance over different substitute CT approaches

Approach category				MRI sequence/contrast		Number	Site	Performance metric				Note	References
*main*	*sub 1*	*sub 2*	*sub 3*					Δ*Dose [%]*	*MAE [HU]*	*DSC*_*bone*_	*Other*		
Voxel	Semi-automatic			T1w		20	brain				1	Δ_ROI_	[61]
	Threshold			dUTE		1	phantom	2		0.81	87	C_bone_	[62–64]
				dUTE		5-19	brain	0.5	230	0.49-0.65	90	C_tissue_	[22, 35, 65]
				Dixon		2	brain				94/77	SS_bone_	[66]
				dUTE	Dixon	6-98	brain			0.75	88/88,81	SS_bone_, C_tissue_	[67–69]
	Probabilistic	Clustering	Fuzzy c-means	T2w	Dixon	2-5	brain				97-98/75-94	SS_bone_	[70, 71]
				T2w	T1w	10	brain				98/74	SS_air_	[52]
				dUTE	Dixon	9	brain		130				[72]
		Bayesian	Markow RF	dUTE		5	brain	1	204-247	0.53-0.59			[22]
	Regression	Discriminant		dUTE	T2w	3	phantom	2.3	88				[73]
				dUTE	T2w	3	brain	1.2	153				[74]
		Gaussian		dUTE	T2w	5-9	brain	0.9-1.5	137-140	0.85	68-94,98	γ_11_, γ_33_	[23, 75–77]
				dUTE		5	brain	1	136-148	0.67-0.72			[22, 48]
		Random F		dUTE		5	brain	1	128	0.74			[22]
		Semi-automatic Dual		T1w	T2w	9	prostate	0.7	75	0.91	99.9	^a^, γ_22_	[78]
				T1w	T2*w	10-15	prostate	0.3-2	135		93	γ_11_	[21, 79, 80]
		PCA		T1w		10	torso				5	Δ_bone_	[81]
	Sinogram			T1w		10	brain			0.85		^b^	[82]
	Neural network			T1w		3	brain			0.78			[83]
				dUTE		4	brain			0.83			[84]
	Pattern recognition			dUTE	Dixon	10	brain				76	C_air_	[85]
	Hybrid	Neural Network	Template	dUTE		4	brain			0.77			[86]
		Gaussian	Spatial info	dUTE	T2w	9	brain		130				[75]
		Random F	Spatial info	T1w		9-10	brain			0.92-0.98		^c^	[87, 88]
Atlas	Pattern recognition	Patch Patch Probabilistic		T1w		5 40	brain	0.5	85	0.84 0.75			[48] [89]
	Deformable			T1w		28	whole body				0.88	DSC_brain_	[90]
				T1w	T2w	5-17	brain	1-2.5	97-114	0.63-0.83	1.7	Δ_ROI_	[24, 48, 91–94]
				T2w		37	prostate	1.5		0.79			[25]
				T2w		10	cervix	0.3					[95]
	Hybrid	Deformable	Patch	T1w		17	brain		101				[96]
				T2w		39	Prostate	0.3	40.5	0.91	100	γ_22_	[97]
Hybrid	Deformable	Probabilistic		T1w		9-27	brain		126	0.86	86/90	SS_bone_	[26, 98]
				T2w		10	prostate	0.2	36.5		99.9	γ_21_	[27]
		Regression	Random F	T2w		20	prostate				0.83	DSC_prostate_	[99]
		Threshold		dUTE		154	brain			0.81			[100]

Column 1: Overall approach: Voxel, atlas or hybrid. Column 2: Sub categories within each main approach. Column 3: MRI sequences/contrasts applied in the studies. Column 4: Number of subjects included in the studies (patients, volunteers, phantoms). Column 5: Anatomical site investigated. Column 6: Performance metrics. Column 7: Specification of “Other” metric and comments. Column 8: References to included studies. SS_x_ = % specificity @ % sensitivity for tissue x, C_x_ = % correctly classified voxel for tissue x, Δ_x_ = mm distance bt. CT and sCT for tissue x, γ_xy_ = % of points with γ_x__%ymm _< 1 where x and y are the dosimetric and geometric deviations, respectively, ^a^MAE of whole FOV and DSC based on 2D DRR, ^b^ Overlap ratio similar to DSC_bone_. ^c^ Bone > 600 HU. Method abbreviations: *Bayesian* Bayesian statistics, *Markow RF* Markow Random Fields, *Discriminant* Discriminant analysis, *Random F* Random Forest, *PCA* Principal Component Analysis, Patch = cluster/collection of MRI voxels, Deformable = deformable registration

### Method categories

The methods were organized with subcategories according to a main voxel, atlas or hybrid approach in Table 1. Voxel based sCT generation utilizes the contrast in the MR image independently of the voxels spatial location. This makes it a potential computational attractive approach. The voxels can be converted into CT numbers in multiple ways which we have subcategorized below.

*Semi-automatic* refers to some kind of manual intervention from the user to make the method work, e.g. delineation of the bone or manually established intensity thresholds below/above which a voxel is categorized into a certain tissue category. *Threshold* covers methods which use the tissue relaxation constant to differentiate between different tissues. For the dUTE sequence/contrast, the T2 relaxation time of the bone (0.5–2 ms depending on magnetic field strength) can as an example be used to categorize voxel which decays > 1/(0.5–2 ms) into bone and voxels which remain constant (or decay slowly) over the short acquisition time into soft tissue [35]. *Probabilistic* refers to methods which can assign a probability of a voxel to belong to different tissue classes with e.g. a corresponding bulk electron density (sCT) or organ label (auto contouring). This could be done by assuming that the MR intensities come from a mixture of K normal distributions (tissue classes) with a corresponding mean and standard deviation. The initial mixture can be estimated with an expectation maximization algorithm through unsupervised training, i.e. an electron density (CT number) is assigned to each tissue class subsequently. For each voxel, a probability can then be calculated for all tissue classes and the voxel can be assigned to the tissue class for which the highest probability was calculated. This is known as *Bayesian statistics. Markow Random Fields* include the tissue classes of neighbor voxels in the probability calculation of a given voxel [22]. Fuzzy c-means clustering use a similar strategy of dividing the MRI voxels into distinct clusters (tissue classes). Cluster similarity coefficients are then calculated for each voxel which is then assigned to the tissue which it resembles the most. A number of different similarity measures exist. *Regression* collects methods which correlate MRI intensities to CT numbers through (statistical) regression on a continuous scale. This can be done in a fashion similar to the Bayesian approach here termed *Gaussian* by including co-registered CT intensities in an initial supervised training phase of the data that establish the prior mixture of the K tissue classes [23]. *Discriminant analysis, Principal Component Analysis* and *Random Forest* are other strategies of performing such a regression between MR and corresponding CT data. *Sinogram* use a forward projection CT like approach to transform the MRI scan into raw MR data where the different tissues are subsequently identified. *Neural network* describes supervised training of a correlation model in a hidden layer with an MRI input layer and a CT output layer. *Pattern recognition* in a voxel setting compares an MRI pattern, e.g. a cluster of 3x3x3 size MR voxels known as a *patch*, with a pre-established correlation between MR patterns and CT numbers obtained through supervised training. Different measures for pattern similarity can be used such as the (normalized intensity) Euclidean distance. *Hybrid* voxel methods combine a voxel based method with somewhat loose information of the voxels location, e.g. distance from center of the brain.

Atlas based sCT generation use the location of an MRI voxel to establish the corresponding CT number by aligning the voxel to an atlas with a pre-known correlation between the MRI voxel location and corresponding CT number. This is potentially more computational challenging as each patient's MRI has to be aligned with an atlas with no possibility of exploiting a pre-training model (except for the atlas building). In an atlas setting, *Pattern Recognition* compares similarities of patient MRI patches (see above) with atlas MRI patches within a limited search volume after alignment. *Deformable* refers to methods which use deformable registration, i.e. non-rigid registration, to assign CT numbers from an atlas to a given MRI scan. The patient's MRI is first registered non-linearly with the atlas MRI. This could be one registration if the atlas consists of one average or template patient. Otherwise, the MRI has to be individually registered to all MRIs in a multi-atlas which is computationally less attractive. Alternative, one can settle with one (entrance) registration if the multi-atlas is internally registered, i.e. a registration map between the entrance atlas and the other atlases has been pre-established. The deformation map between the patient and atlas MRI is then applied to the corresponding atlas CT and the sCT produced. If multiple atlases are used, a fused CT number can be applied.

*Hybrid* atlas methods combine multiple methods within the atlas based category. This could be a method which combines deformable registration with a (patch) pattern recognition approach to minimize the influence of registrations which resulted in a poor alignment.

**Fig. 5:**
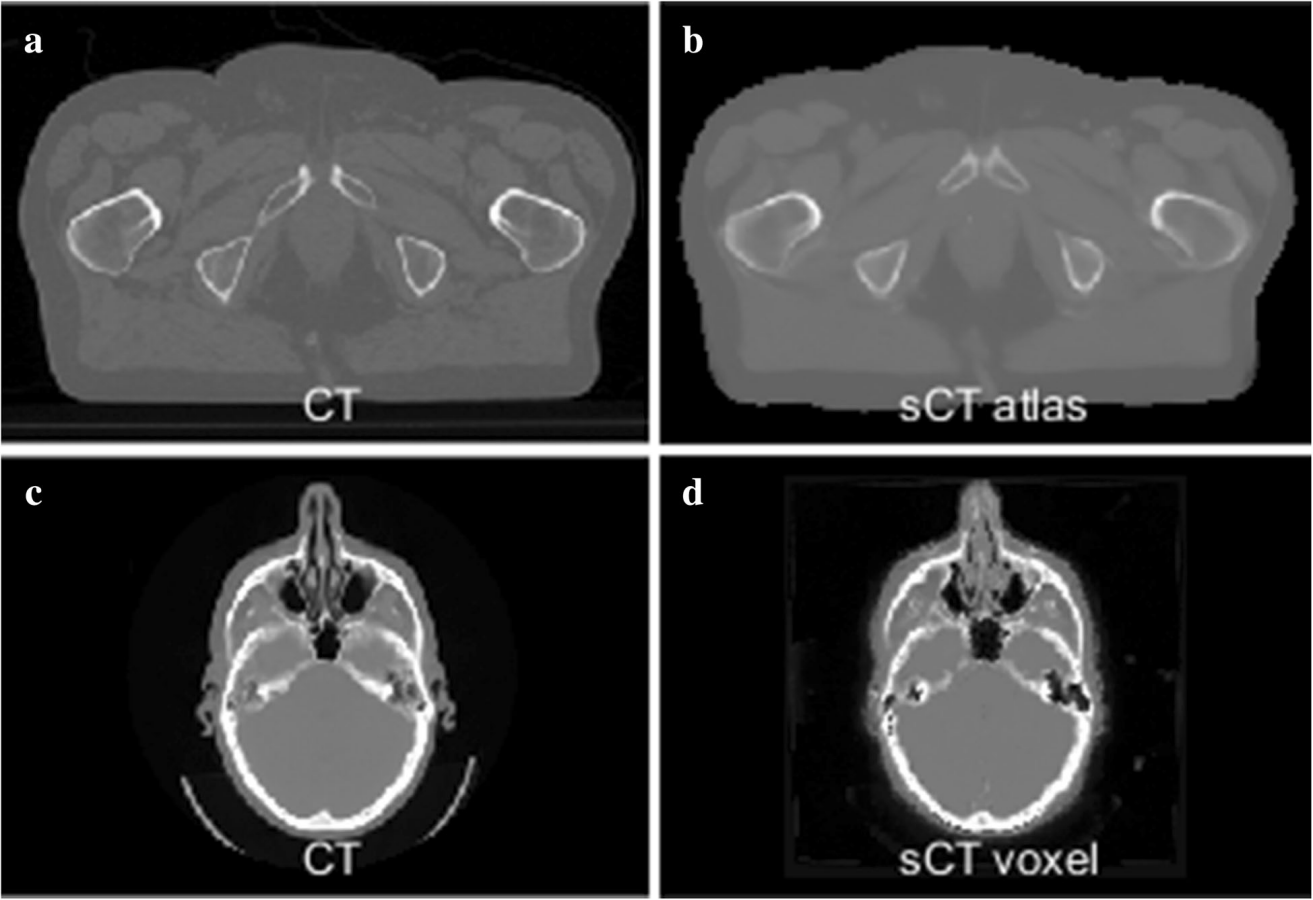
Examples of sCT generation for the pelvic (*top*) and brain (*bottom*). **a**: An axial CT slice of the pelvic from a prostate patient. **b**: The corresponding sCT slice created with an atlas patch based approach [49]. **c**: An axial CT slice of a brain patient. **d**: The corresponding sCT slice created with a voxel Gaussian mixture regression based approach [75]

The final *Hybrid* approach combines categories of the voxel and atlas based approaches. This could for example be a calculation of two probability density functions (PDFs) for each voxel; one based on deformable registration (atlas) and the other based on Bayesian statistics (voxel). The two probabilities are then combined into a unified posterior PDF which determines the final assignment of CT number to the voxel [26]. An example of atlas and voxel based sCT generation for the pelvis and brain can be seen in Fig. 5.

### Performance metrics

**Fig. 6:**
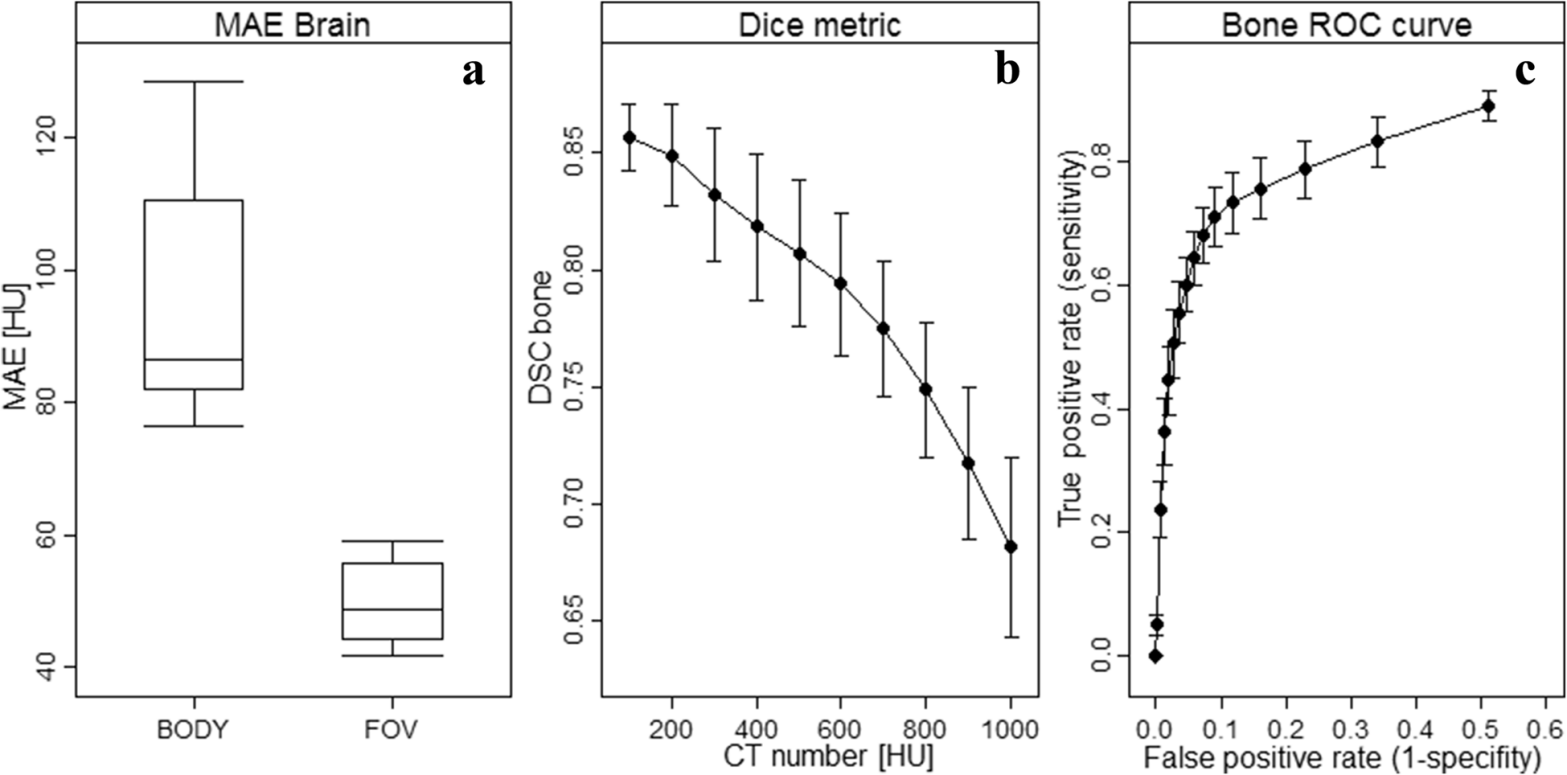
Performance metrics dependence on the region of interest and CT threshold number for the bone. All metrics are calculated on substitute CTs from 3D T1w MR images of 6 brain patients [47] using an atlas patch based method [48]. **a**: The *boxplot* shows the MAE within the body outline (*left*) and the whole field of view (FOV, right). The medians were 87 and 49 HU for the body and FOV MAE, respectively, and were significantly different (*p* < 0.002). **b**: The Dice similarity coefficient (DSC) metric for bone as a function of threshold CT number. **c**: Receiver operating characteristic (ROC) curve of the sCT bone as a function of CT threshold number (thres). True positive (TP) = sCT > thres & CT > thres, false positive (FP) = sCT > thres & CT < thres, true negative (TN) = sCT < thres & CT < thres and false negative (FN) = sCT < thres & CT > thres. Sensitivity = TP/(TP + FN) and specificity = TN/(TN + FP). The threshold was varied from 100 (*right*) to 3000 (*left*) HU in steps of 100 HU. Only voxels > 100 HU on the CT, i.e. the bone region, was included in the evaluation to keep the TN number (non-bone tissue on the sCT and CT) to a reasonable number

Three common metrics reported in the literature to score the performance of a given sCT generation method were chosen. The first metric, *ΔDose,* describes the dosimetric agreement when performing dose calculations on the sCT as compared to the standard CT. This is usually quantified as the percentage difference in either single characteristic points, e.g. iso-center or dose prescription point, or in dose volume histogram (DVH) points. The general equivalent uniform dose (gEUD) has also been used to describe the biologically relevant differences of the entire DVH. Another commonly reported metric for quantifying dosimetric differences is the gamma index [36]. This metric covers spatially correlated dose deviation in both the high and low dose regions. Whenever differences in multiple dose metrics were reported, e.g. multiply DVH points, a collectively representative value, e.g. the mean of all the deviations, was chosen. The second metric is the mean absolute error (MAE) and describes the absolute voxel-wise difference in HU defined as

$$ M A E=\frac{1}{N}{\displaystyle \sum_{i=1}^N}\left| C{T}_i- sC{T}_i\right| $$

where *N* is the number of voxels, *CT* is the standard CT and *sCT* is the substitute CT. This metric is typically lowered as the number of voxel similar to water or air increases, e.g. moving from the brain to the pelvis or including air outside the body outline (see Fig. 6a). The MAE shows a great variation over the different tissue regions. For the data presented in Fig. 6a, the shown median MAE of 87 HU for all tissue in the skull covers a median MAE of 216, 36 and 261 HU for air, soft tissue and bone, respectively.

The third metric, the dice similarity coefficient (DSC) for bone [37], is a geometric score describing the overlap between the CT and sCT bone volumes. It is defined as

$$ D S{C}_{bone}=\frac{2\left({V}_{C T}{\displaystyle \cap }{V}_{sCT}\right)}{V_{C T}+{V}_{sCT}} $$

where *V* is the volume of the bone on the CT and substitute CT (sCT), respectively. A similar measure, the Jaccard coefficient (JAC) can be converted to the DSC through the relation DSC = 2 · JAC/(1 + JAC) [38]. Another commonly reported metrics is the sCT specificity and sensitivity for different tissues such as the bone. The bone DSC, sensitivity and specificity will depend on the threshold value set for the CT number of the bone (see Fig. 6b and c). Other metrics are otherwise noted in the legend of Table 1.

The performance metrics reported in the literature cannot be directly compared due to issues such as patient selection and exclusion criteria which are often underreported and can introduce a bias. Further, the amount of preprocessing included in the algorithm such as data normalization and bias field correction will affect the final result. Still, methods performing equally well in the same body region should produce performance metrics within the same gross interval.

## Results

The statistics reported in Fig. 2 and Fig. 3 indicate that most studies are carried out on the brain for 5-10 patients with PET/MR applications in mind using a voxel based method. It is common to use model input data that coincide with the image data used for the delineation of the target volume or organs at risk. The main benefits are avoidance of unnecessary registrations in the workflow to compensate for intra examination patient motion and to keep the examination time as short as possible. Therefore evaluation of T1w input data is common for the brain region, while T2w input data is common for the pelvic region. dUTE has the benefit of enabling separation of cortical bone and air, but has not been reported useful for delineation purposes. Our review shows that at present point dUTE has only been evaluated for intra-cranial conditions or phantoms (Table 1). Dixon based sequences enable separation of water and fat signal, and is currently often used for attenuation correction of PET data in hybrid PET/MR scanners. Dixon sequences also tend to be fast and the in-phase sequence has been reported useful for identification of fiducial makers in the prostate [39]. Table 1 shows a wide diversity in terms of the methods and MRI sequences investigated in the literature. Overall, the dosimetric agreement is in the order of 0.3–2.5%. A strict gamma criterion of 1% and 1mm has a range of passing rates from 68 to 94% while less strict criteria show pass rates > 98%. Given the relatively small order of dosimetric disagreement, the residual distortion (i.e. remaining distortion after applying distortion correction procedures) present in the MR and hence sCT images subject to dose calculations seem to be of minor importance in order to reach an acceptable dosimetric accuracy. Rather, these seem to be more critical for accurate target and OAR delineation [40]. The MAE is between 80 and 200 HU for the brain with a majority of values lying in the 120–140 HU interval. The MAE values are around 40 HU for the prostate (pelvis region). The Dice score for bone is between 0.5 and 0.95 across the different methods, MRI sequences/contrasts and anatomical sites. The specificity and sensitivity range from 75 to 98%. As is apparent from Fig. 6c, increasing the specificity will decrease the sensitivity and vice versa. A compromise seems to be in the upper 80s for both quantities. Correctly classified voxels average around 84% for the different methods. No strict relationship between the dosimetric and geometric agreement as scored by the metrics common in the literature is present. Further, when a large number of patients are present in a study, i.e. more than 20 patients, the dice score seem to be in the lower range (<0.85) of the reported values probably caused by a larger diversity in the patient material.

## Discussion

Table 1 is not a complete list of all methods used for generating a sCT, due to the search strategy, time of search and exclusion criteria applied, some novel methods such as sparse representation [41] and Random Forest with auto-context modelling [42, 43] are not included. With these limitations in mind, our investigation shows that sCT generation for MRI-only based radiotherapy or PET/MRI attenuation correction seems to be a comprehensively tested area of research given the variety of investigated methods which are able to produce a sCT from an MRI scan. This presents an advantage in the sense that a broad material is currently available to further develop on. This strength, however, also presents a challenge for the field. There is no obvious method or MRI contrast(s) which seem to be clearly favorable from the others and hence no clear indication as to which path of promising methods to pursue. The data in Table 1do not indicate that inclusion of more MR contrasts in the generation of the sCT automatically increase the accuracy. This is encouraging as extra sequences both increase the total acquisition time and overall complexity of the method and the workflow. Notably, the dice score of the voxel hybrid approaches using Random Forest (Random F) in Table 1 creates an almost perfect overlap between CT and sCT bone volumes especially considering the high threshold of 600 HU applied in these studies (see Fig. 6b). These reported results should encourage other researchers to reproduce this method on different datasets especially since a pure Random F approach on dUTE images does not produce similar high performance scores. The average dosimetric deviations reported in Table 1 should be used with some caution. Korsholm et al. reported on dose deviations using bulk density corrections for multiple treatment sites [44]. With a bulk density correction for the bone, the average deviation for median PTV dose of the prostate was practically zero but covers a range of -1.1 to 1.1% representing the 95% confidence interval. They further argued that the 95% confidence interval should be within a 2% dosimetric deviation to produce clinical acceptable results.

The focus of the present review is the conversion of image data acquired with MRI to electron density or HU maps to facilitate a so called MR-only treatment planning workflow. In addition and not limited to the MR-only approach, use of MR in radiotherapy requires MR data with a minimum of geometrical distortions. It has been shown that distortions due to non-linearity in the spatial encoding gradients can be successfully corrected using deterministic algorithms [45], and chemical shift artifacts and distortions caused by susceptibility effects can be minimized using a sufficient bandwidth [46]. There is, however, still a need to further confirm these results and develop efficient quality control techniques, but this is out of the scope of the current review.

### Standardization

One could argue for a need to unify the efforts to localize promising candidate for sCT generation. A possible way is to standardize the calculation of the performance metrics reported in the literature. It is clear from Fig. 6 that the MAE, DSC_bone_, specificity and sensitivity metrics have their limitations and further do not necessarily reflect the corresponding dosimetric performance of the method. Quantitative metrics that more unambiguously reflect a correlation between the geometrical and dosimetrical agreement are needed and these should further display an independence of parameters such as selected CT number threshold and field-of-view. An example could be to score the sCT-CT difference in bins covering the HU scale independent of the number of voxel present in each bin [47]. Another example could be to use differences in radiologic (water equivalent) path lengths which represent both a geometric and dosimetric property [48, 49]. The accuracy of the performance metric should further be related to the application in question, e.g. RT or PET/MRI. To benchmark results, another possible way would be to make datasets consisting of MRI scans with a variety of contrasts and corresponding CT scans for different anatomical sites public available. Such a dataset currently exists for quantitative imaging of biomarkers [50] and a similar dataset for sCT generation could serve as a mandatory step for method benchmarking before publication. Issues related to the algorithms processing of MRI signal normalization and correction would further become apparent on such a dataset. Currently, many studies only benchmark the sCT against the gold standard CT. In addition, a dosimetric comparison between a proposed method and the investigated MRI scan(s) set to water should be included in a study to give a perspective of the reported quantities. Another issue is a number of relevant items which are often not addressed. These cover inclusion or exclusion criteria for the investigated patients which could create a bias, computation time needed for a given method to work and restrictions in the possible clinical implementation of the method.

### Clinical implementation

So far, the authors are aware of two institutes which currently have implemented in-house developed MRI-only methods clinically. The first institute use a voxel based dual regression approach on the prostate and have currently treated around 150^2^ patients [21, 51]. The second institute use a voxel based probabilistic approach with fuzzy c-means on the brain and have currently treated around 30 whole brain and 15^3^ focal brain cases, respectively [52]. Further, commercial solutions for sCT generation are becoming available [27, 53]. Stereotactic radiosurgery treatment planning of brain tumors have been carried out on MRI scans set to water for decades [54]. Brain radiosurgery was outside the scope of this review but one could question this practice given published literature which demonstrates an improvement in dosimetric accuracy as compared to a pure water based dose calculation for the brain, see e.g. [55]. For the brain and other treatment sites, it has further been shown that a bulk density assignment is probably sufficient for RT treatment planning [44, 56].

Treatment delivery and quality assurance (QA) of an MRI-only based RT workflow are other important issues which need to be addressed for clinical implementation of MRI-only RT. In terms of commissioning an MRI-only workflow, the guidelines as to what tolerances which are acceptable are limited. As mentioned earlier, a 2% dosimetric agreement between sCT and CT based dose calculation seems to be of an acceptable order [44] and most of the investigated methods demonstrate an agreement better than this. For kV X-ray based image-guided RT (IGRT) delivery systems, the cone beam CT (CBCT) seems to provide an acceptable solution for patient setup of both brain and pelvic patients (marker match is probably sufficient for prostate) [47, 57, 58]. The CBCT can further be used for patient specific QA of the generated sCT as it provides an independent estimate of the CT numbers [47]. sCT QA verification efforts using the electronic portal imaging device (EPID) has also been proposed [53]. All of the above elements should be considered when formulating an MRI-only based RT protocol.

## Conclusions

In summary, a variety of promising approaches for substitute CT generation exist which seem to provide results acceptable for clinical implementation. This also includes methods based on clinical simple standard MRI sequences/contrasts. However, the field suffers from a current lack of an established benchmarking method and reporting consistency, which challenge the commitment from interested vendors and hence risk a delay for a broad clinical implementation.

## Footnotes

1 In photon-based RT, the main interest is to convert the CT number (HU) into an electron density (relative to water) for dose calculation purposes. Therefore, CT number and electron density is used interchangeable throughout the text.

2 Korhonen J. Personal correspondance. 2015.

3 Balter JM. Personal correspondance. 2015.
